# Toxic effects of formaldehyde and the protective effect of docosahexaenoic acid in *Drosophila*

**DOI:** 10.1515/tnsci-2020-0186

**Published:** 2021-10-04

**Authors:** Yanli Hua, Chao Ma, Shuyi Huang, Ruomeng Wang, Jian Chen, Qing Guo, Jiaojiao Zhou, Hemin Zhu, Wenjie Li

**Affiliations:** Department of Human Anatomy, Medical College of Soochow University, 199 Ren-Ai Road, Suzhou 215123, PR China

**Keywords:** formaldehyde, docosahexaenoic acid, neuroprotection

## Abstract

Formaldehyde (FA) is a commercially important chemical applied in industry and scientific research. However, FA has a distinct impact on learning and memory. Although the mechanisms of FA toxicity have been well studied, additional research is required to establish the mechanisms of neuroprotection in cases of FA exposure. Docosahexaenoic acid (DHA) is a polyunsaturated fatty acid with a variety of health benefits, including the enhancement of learning and memory. In this study, we investigated the neuroprotective effects of DHA in *Drosophila melanogaster* that had ingested FA. Our data suggested that DHA enhanced reproductive processes, leading to an increase in the number of eggs, larvae, and adults. Surprisingly, we found that DHA had a mild protective effect against FA-induced impairments in learning and memory.

## 1 Introduction

Formaldehyde (FA) has many industrial and scientific applications. It is used as a preservative and an antimicrobial agent in both skincare products and within industrial production, where it is sometimes used as a raw material for production [1]. FA is a common chemical activator found in paint, clothes, medicine, and industrial products, and is a component of diesel and gasoline exhaust [2]. Moreover, it has been widely used for the preservation of cadavers for teaching purposes in medical colleges. However, the levels of FA in a university dissection room (0.36 ppm) were found to exceed the established safety threshold [3]. Such exposure may increase the risk of health problems among lecturers in anatomy departments.

Some commercial products that employ the preservation effects of FA do not contain FA themselves, but have agents that release FA under specific usage conditions, termed “FA releasers.” FA is also a common cause of contact allergy [4], which is associated with childhood asthma [5], and potentially alters signaling pathways associated with cancer, inflammatory response, and endocrine system regulation [6].

The effect of FA administration on *Drosophila melanogaster* (fruit fly) has been studied for more than half a century. Researchers have examined FA-induced changes in DNA replication [7], the mutagenic action of X-rays [8], gene mutations [9], the molecular consequences of the alcohol dehydrogenase gene [10], and protein–DNA crosslinking [11]. However, the impact of FA exposure on reproduction, motor ability, lifespan, learning and memory in *Drosophila* is not completely clear. Potential mechanisms underlying FA-induced reproductive and developmental toxicities, including chromosome and DNA damage (genotoxicity), oxidative stress, altered level and/or function of enzymes, hormones and proteins, apoptosis, toxicogenomic and epigenomic effects (such as DNA methylation), were identified. A systematic review by Duong et al. showed that a strong association between both reproductive and developmental toxicity and FA exposure, at multiple doses and routes of exposures in various species [12]. Hydrogen sulfide (H_2_S), the third gasotransmitter, is an endogenous neuromodulator, which facilitates the induction of hippocampal long-term potentiation (LTP), involving the functions of learning and memory. A study by Tang et al. [13] indicated that FA impaired learning and memory by disturbing the generation of endogenous H_2_S in the hippocampus, ultimately leading to oxidative stress-mediated neuron damage. Furthermore, Tong et al. [14] reported an association between increased endogenous FA levels and abnormal spatial memory, which appeared to be caused by a decline in global DNA methylation due to interference from DNA methyltransferases (DNMTs). Lu and colleagues [15] also demonstrated that FA inhalation negatively affected spatial learning and memory in mice, presumably due to neuronal damage resulting from oxidative stress.

Based on the toxicity associated with FA, the European classification, labeling, and packaging of FA states that it is a human carcinogen (Group 1B and mutagen 2) [16], and the U.S. Environmental Protection Agency classifies FA as a probable human carcinogen [17]. However, no drugs or nutrients that alleviate the effects of FA overexposure have been identified. Thus, there is a need for re-evaluation of the risks associated with FA exposure in occupational settings, as well as the examination of compounds that could have a protective effect, particularly against FA-induced alterations in learning ability.

Docosahexaenoic acid (DHA) is a long-chain polyunsaturated fatty acid that is ample in fish oils. DHA is essential for the growth and functional development of the brain in infants. It has a variety of health benefits, including enhancing visual ability, facilitating cognitive activity including learning and memory, and reducing neurodegeneration [18]. DHA is fundamental to the formation and function of the nervous system and is particularly important for brain and retinal function in humans [19]. Further, dietary DHA modulates the maturation and survival of photoreceptor cells. The beneficial effects of DHA on visual function have been well established. Notably, Shindou et al. [20] reported that retinal DHA maintains the disc shape of photoreceptor cells. Moreover, DHA has been found to boost increases in cognitive ability [21].

Accordingly, Jiang et al. suggested that DHA supplementation could be used to address cognitive dysfunction [22]. Further, DHA may play protective roles against the effects of neurodegenerative disease. For instance, Parlak et al. reported that DHA treatment protected dopaminergic neurons in the substantia nigra by increasing the phosphorylation of nNOS at serine 852 in a model of Parkinson’s disease [23]. Furthermore, DHA has been found to modulate Aβ aggregation by stabilizing soluble fibrillar Aβ oligomers and then reducing the formation of both Aβ plaques and prefibrillar Aβ oligomers [24].

Regarding the relationship between DHA and measures of learning and behavior, low brain DHA has been associated with behavior modification as well as impaired learning and memory [25]. Correspondingly, Hashimoto et al. found that DHA-administered rats had a higher level of fear-related avoidance memory [26].

With the increasing application of FA in industry and scientific research, many researchers have examined the effects of FA toxicity on reproduction [27,28], learning, and memory [13,15,29,30]. However, the mechanisms by which DHA might counter FA-induced changes in reproduction and learning, and memory are unclear.

To address this in the present study, we initially counted the pupal and offspring number, the weight of the adult fly. In addition, in order to comprehensively confirm various damaging effects of FA on *Drosophila* and the protective effect of DHA, we also tested climbing assay and lifespan to study the toxicity on motor ability and development of FA. Secondly, we studied the neurotoxicity of FA by using learning and memory assay, since the brain-derived neurotrophic factor (BDNF) is a widely present neurotrophic factor in the central and peripheral nervous system, which plays an important role in supporting the survival of existing nerve cells, promoting the generation of new nerves and synapses [31,32]; checking BDNF level by Western blot can evaluate that nervous system function of *Drosophila*. Finally, we evaluated whether DHA showed protection against FA-induced toxicity with the same assay. We found that DHA showed anti-toxicity on development and motor ability and neuroprotection on learning and memory in our *Drosophila* model.

## 2 Materials and methods

### 2.1 Fly stocks

All *Drosophila* stocks (19B03-Gal4) were maintained with a 12 h light/dark cycle at 25°C and 55% humidity, raised in noncrowded conditions on a standard cornmeal medium. Fly food consisted of (per 1 L) 10 g agar, 7.25 g sucrose, 30 g glucose, 24.5 g yeast, 50 g cornmeal, 17.5 mL methyl 4-hydroxybenzoate, 4 mL propionic acid. The following *Drosophila* strains were used: control (Bloomington Stock Center).

### 2.2 Culture medium with FA and DHA

For FA (and DHA)-containing standard cornmeal–yeast–agar medium, FA (and DHA) was added when the culture medium approached 50–60℃; then, the indicated volume of FA (and DHA) was pipetted quickly and then mixed thoroughly with a stirring rod before the medium solidified. Given that DHA breaks down slightly under the light condition, the newly containing-FA (and DHA) was kept at 4℃ black conditions. The prepared medium was kept less than 3 days before the experiment was carried out. Table 1 shows the gradient concentration of FA and DHA.

**Table 1:** FA or FA + DHA concentration in the medium

	Control group	FA group	FA + DHA group			
FA (%)	0	0.150	0.150			
DHA (µL/100 mL)	0	0	1	2	3	4

### 2.3 Pupal and adult fly counting

Ten new eclosed adult males and 10 virgin females were separated into fresh food vials for 3 days. Then, all parental flies (F0) were removed after crossing for 24 h. Next, the pupal climbing on the vial and the number of eclosed adult fly (F1) were recorded every other day until no adult fly was eclosed.

### 2.4 Adult fly weighing

In the method “Pupal and adult fly counting” above, F1 males and females were collected and separated into several fresh food vials for 3 days; then, 20 flies (male or female) as one unit were weighed, and the weight were recorded. Each indicated group included at least 3 different units.

### 2.5 Lifespan assay

Adult male flies were used for survival analyses. Flies were grown at 25°C and moved to fresh medium every 4 days. Death was recorded every other day until all flies from the experimental groups were dead. Log-rank tests were performed for statistical analysis via Prism GraphPad software.

### 2.6 Adult climbing assay

Adult male flies of 3–4 days age were put into a 50 mL measuring cylinder and three negative geotaxis tests were carried out with 20 flies in each group. The flies were gently tapped to the bottom of a vial and allowed to climb for 30 s. The climbing ability of flies was quantified as the number of animals that reached the 10 cm of the cylinder in 15 s. The test was repeated 3 times for each group. The number of flies that reached the 10 cm of the cylinder was converted into a rate value.

### 2.7 The general procedure of the learning experiments

The larvae used in the experiment underwent the essential mid-third instar transition from foraging (feeding) to wandering (nonfeeding) behavior before pupariation and metamorphosis. This transition is critical for reward learning because wanderers have reduced motivation for feeding and might not perform optimally in feeding-related tasks [33,34].

Based on this principle, larvae that were active on the surface of the culture medium but not yet climbing up the tube wall were selected as the experimental subject. For hungry larvae, food should be a powerful reward. In this study, the larvae were placed in a sweet medium with a neutral odor X (experience denoted as X+), and then transferred to a sugar-free medium and exposed to another neutral odor Y. After repeated training, the larvae were expected to establish a conditioned reflex. Specifically, the larvae were expected to associate odor X in the presence of sugar. Thus, when the larvae were exposed to both odors in the absence of sugar, they were expected to travel toward the odor that they associated with a reward.

In this experiment, FRU (fructose, F9048, Sigma) was used as the rewarding stimulus, and AA (amyl-acetate, A0021, TGI) at a 1:50 dilution and OCT (1-octanol, 297,887, Sigma) were used as odors with neutral biological potency.

In the training trials, larvae were trained using two reciprocal training regimens. First, the animals received stimulus X with a positive reinforcer (+) and stimulus Y without a reinforcer (Train: X +//Y; the chemical identity of X: AA, Y: OCT, reinforcer: fructose). In the second regimen, the animals received stimulus Y with a positive reinforcer and stimulus X without a reinforcer (Train: X//Y+). The 2 M FRU reward was added to the agar on the bottom of the positive reinforcer perish dish when preparing the test. Immediately before each trial [35], two pieces of double-sided tape were positioned on opposite sides of the interior surface of a perforated Petri dish lid. Strips of filter paper were placed on both pieces of tape. Twenty microliters of AA (diluted 1:50) or OCT was pipetted onto both pieces of filter paper. In the training trials, the animals were transferred to one of the two training Petri dishes, and the lid was closed. After 5 min, the animals were transferred to the other dish. This training cycle was repeated 3 times. Fresh Petri dishes were used for each trial. After training, the animals were expected to associate one stimulus with a reward and the other with no reward.

In the test trials, the larvae were placed in the middle of the test Petri dish. The test Petri dish did not contain the fructose reward. Two pieces of filter paper were placed on opposite sides of the Petri dish, 5 mm from the edge. Each piece of filter paper was loaded with a different stimulus to create a choice situation, i.e., the container was loaded with stimulus X on one side and stimulus Y on the other side (Test: X–Y).

The number of animals on the X-side, Y-side, middle strip at 3, 5, and 8 min was counted. Then, the preference score (PREF) was calculated as follows:

(1) \text{PREF}\hspace{.25em}\text{X}+\text{//Y}=(\#\text{X}-\#\text{Y})/\#\text{Total}.]

In this equation, # indicates the number of larvae observed on the respective half of the test dish.

Then, another group of 20 animals was trained in a reciprocal manner, and the PREF score was determined as

(2) \text{PREF}\hspace{.25em}\text{X//Y}+=(\#\text{X}-\#\text{Y})/\#\text{Total}.]

We were not just interested in whether learning took place, but also in the degree to which the larvae learned to associate the reward with the stimulus. Learning index (LI, equation (3)) was used to quantify the difference in PREF scores between the X-rewarded and Y-rewarded animals (LI index was divided by 2 to ensure that the LI scores varied between –1 and 1) [31]. The equation for this calculation is as follows:

(3) \text{LI}=(\text{PREF}\hspace{.25em}\text{X}+\text{//Y}\mbox{--}\text{PREF}\hspace{.25em}\text{X//Y}+)/2.]

LI is used to indicate the learning and memory ability of larvae. A higher value indicates better learning and memory ability.

**Figure 1:**
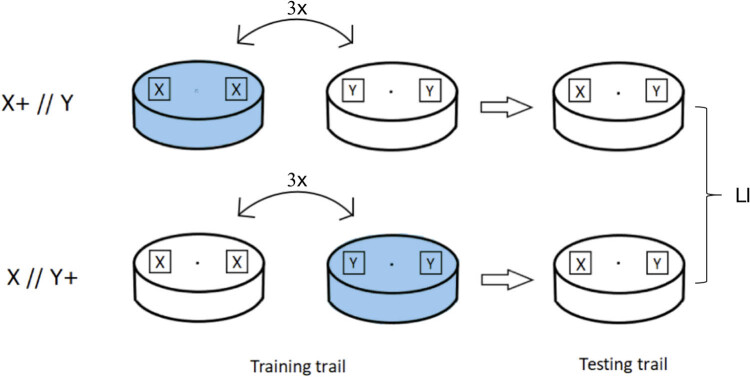
The process of larval training and testing.

Figure 1 shows the method of operation.

### 2.8 Western blotting

To extract protein from whole heads, samples must be kept as much as possible on dry ice until extraction actually begins. The head of *D. melanogaster* was cut-off with a blade, put in a 15 mL test tube, and stored in ice. The frozen heads of the *Drosophila* were homogenized in modified RIPA buffer followed by centrifugation at 1,000×*g* for 5 min at 4°C to remove nuclei and intact cells. Then, centrifuge at 4°C for 20 min at 12,000×*g*, and the resulting supernatant was collected. The protein concentration was determined using the Bradford method. Proteins were separated via standard 10% sodium dodecyl sulfate polyacrylamide gel electrophoresis (SDS-PAGE) and transferred to nitrocellulose membranes. Then, membranes were blocked by 5% skimmed milk in TBS buffer containing 0.2% Tween 20 (TBST) at room temperature for 1 h to avoid nonspecific binding sites. We used 1:150 dilution of polyclonal rabbit anti-BDNF (Santa Cruz; 1:500) to react with the membrane overnight, and 1:500 dilution of anti-β-tubulin antibody (Sigma; 1:5,000) was used as an internal control. After incubation, the membranes were washed with TBST and were then incubated with a horseradish peroxidase-conjugated anti-rabbitIgG secondary antibody (Boster, 1:2,000) in 5% nonfat dry milk in TBST. After washing, the blots were visualized using enhanced chemiluminescence (ECL, Thermofisher). Image J was used to analyze the gray value of the band, and β-tubulin was used as a reference to standardize the amount of BDNF protein in each group. The results were analyzed using GraphPad Prism (Version 9, GraphPad Software, San Diego, CA, USA).

### 2.9 Data analysis

GraphPad Prism 9.0.0 program was used for statistical analysis of data presented as mean ± SEM. The data were analyzed using unpaired *t*-tests, one-way ANOVA followed by Tukey’s *post hoc* test and Mann–Whitney *U* test. The value of *P* < 0.05 was set as the significance level.

## 3 Results

### 3.1 Reproduction and development

**Figure 2:**
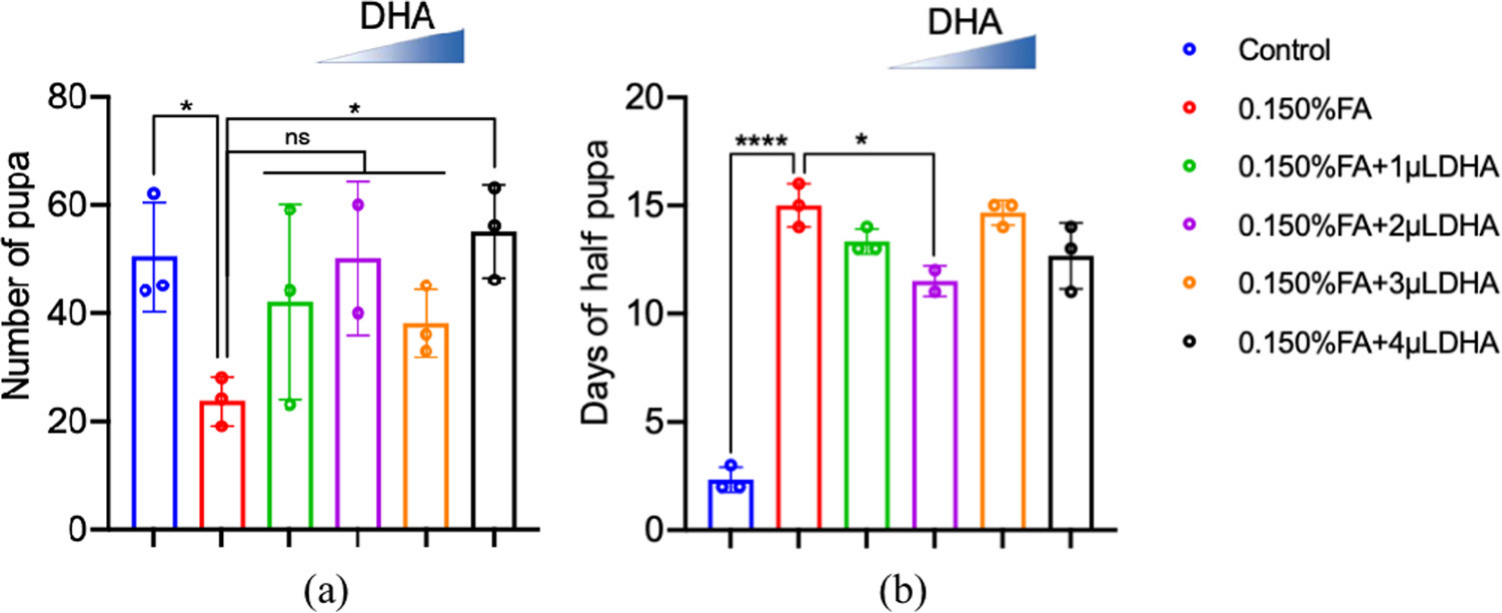
DHA can reverse the reduction of pupa number and alleviate the shortened time from egg to pupa induced by FA. (a) The numbers of pupae were counted from at least three independent experiments. (b) The time from egg to half of the total number of pupa was measured. ns: no statistical significance, *P < 0.05, ****P < 0.0001, *t*-test, one-way ANOVA, Graphpad Prism 9.0.0.

The total amount of pupa and adult flies were counted until it is ensured that no new pupa or adult flies were produced. It took a total of 20 days to record pupal production and 27 days to record adult flies production. Figure 2(a) shows the characteristics of *Drosophila* spawning. The results showed that the fecundity of the *Drosophila* in the 0.150% FA group was significantly lower than that in the control group (*P* < 0.05). The addition of different concentrations of DHA to the 0.150% FA group increased the egg production to different degrees, and the egg production of the *Drosophila* in the 0.150% FA + 4 µL DHA group was significantly increased (*P* < 0.05).

Exposure to FA and/or DHA not only affected the number of eggs produced and the number of flies that emerged but also influenced the rate of growth and development of offspring. The rate of growth and development of the next generation was calculated in terms of the time taken to develop half the number of total pupae, i.e., the day at which half the total expected number of pupae was observed. The results are shown in Figure 2(b). Compared with the control group, the time taken for the larvae in the 0.150% FA group to start to climb the culture tube wall was significantly prolonged (*P* < 0.0001). However, compared with the 0.150% FA group, the time taken for the larvae to start climbing the tube wall was shortened according to the concentration of DHA exposure, such that the time required for the larvae in the 0.150% FA + 2 µL DHA group was significantly shortened (*P* < 0.05). This indicates that 0.150% FA exposure could delay the development of *Drosophila* and that DHA could counteract this phenomenon.

**Figure 3:**
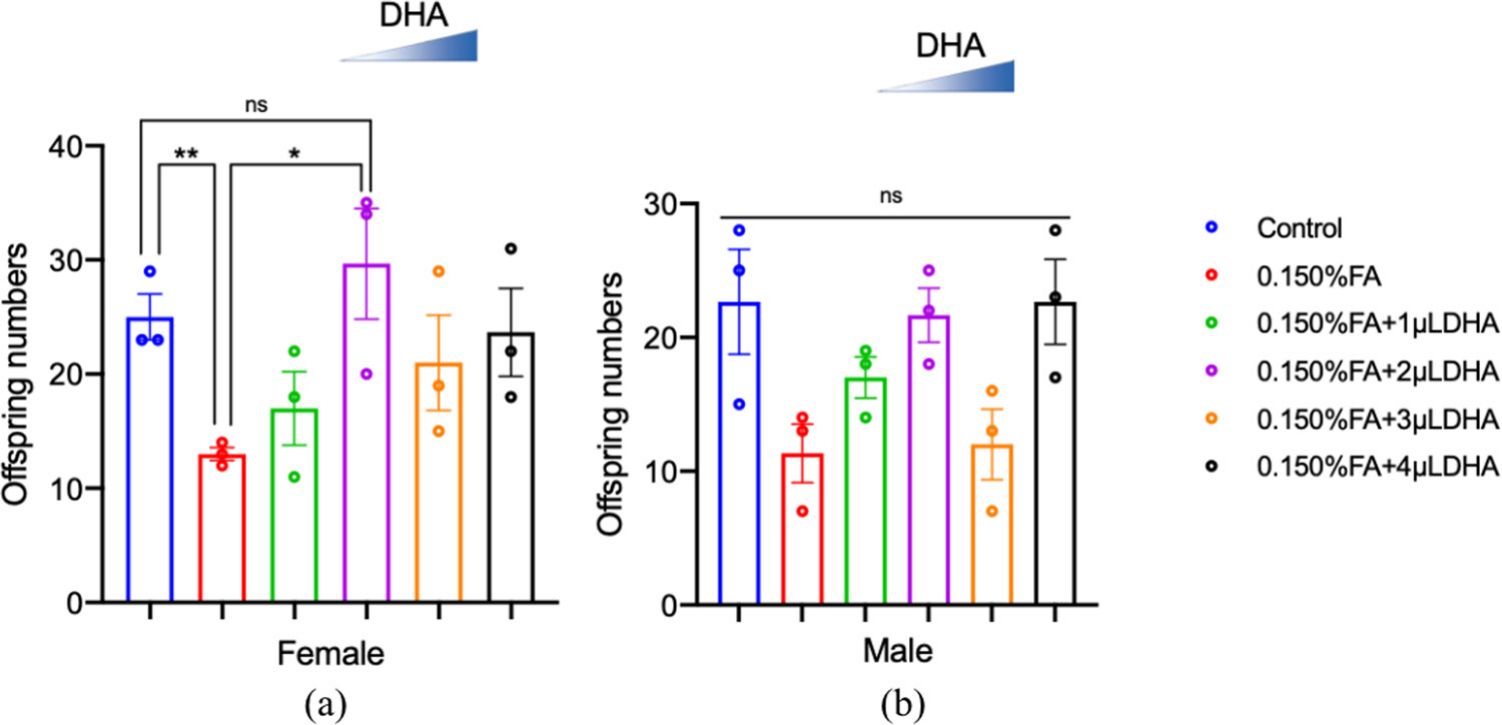
DHA can rescue the reduction of FA-induced offspring. (a) The numbers of offspring of female were counted from at least three independent experiments. (b) The numbers of offspring of male were counted from at least three independent experiments. ns: no statistical significance, *P < 0.05, **P < 0.01, *t*-test, one-way ANOVA, Graphpad Prism 9.0.0.

Figure 3 shows the hatching characteristics of the *Drosophila* pupa. We compared the total number of female and male *Drosophila* in the F1 generation in each group. In the case of F1 female *Drosophila*, the number of female *Drosophila* that hatched in the 0.150% FA group was significantly lower than that in the control group (*P* < 0.01). Compared with that in the 0.150% FA group, the number of F1 generation female flies increased according to the concentration of added DHA, such that the 0.150% FA + 2 µL DHA group had significantly higher proportions of female flies (*P* < 0.05). For F1 male *Drosophila*, there was no significant change.

**Figure 4:**
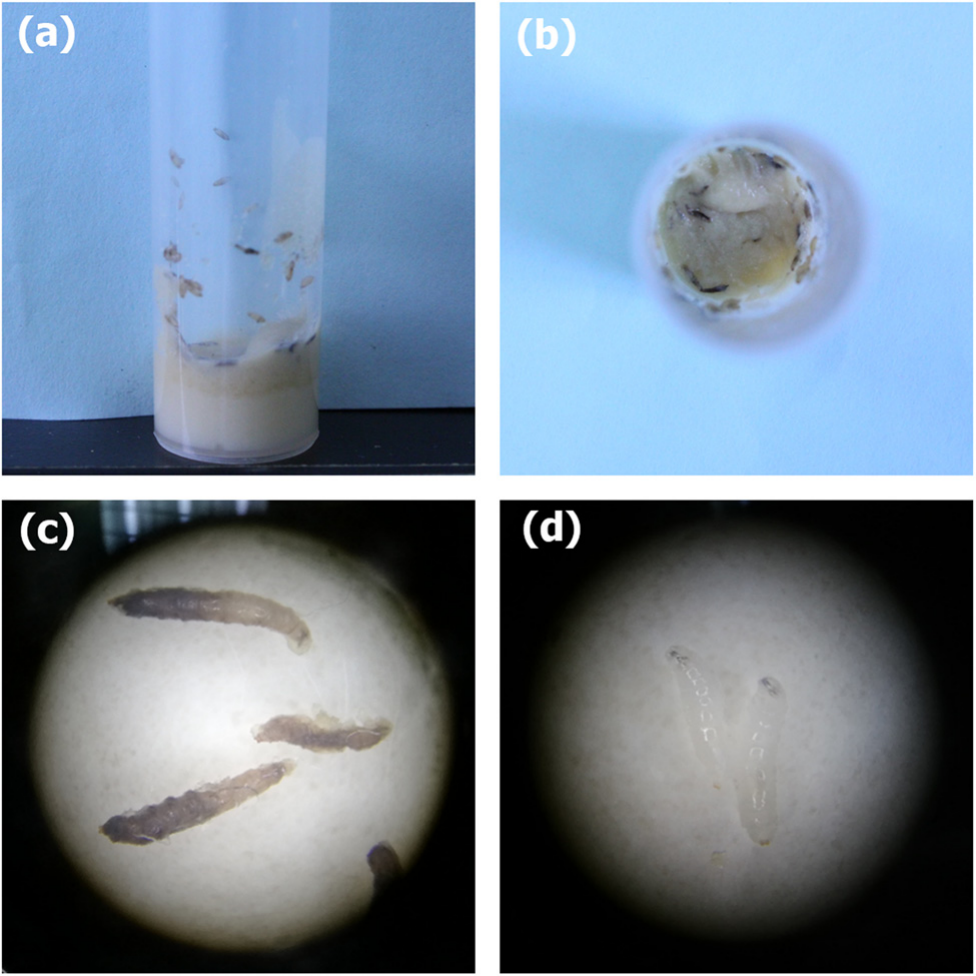
Phenotypic characterization of FA or FA + DHA-induced larvae or pupae. (a and b) In the 0.150% FA group, some of the larvae in the culture tube were black, hard, and dead. (c) Magnified image of the blackened larvae in the 0.150% FA group. (d) In the magnified image, the larvae from the 0.150% FA + 4 µL DHA group were white, soft, and normal in size.

In terms of the morphology of *Drosophila* development, in the 0.150% FA group, a proportion of the larvae were black, hard, and did not develop, indicating that the pupae had died. Figure 4(a) and (b) shows the morphology of blackened and dead larvae in the culture tube, and Figure 4(c) shows the morphology of the blackened larvae viewed through a microscope. However, when DHA was added to the eggs treated with 0.150% FA, this phenomenon disappeared. Figure 4(d) shows the morphology of larvae in the 0.150% FA + 4 µL DHA group on the 7th day of development. The morphology, size, and color of the larvae were normal, indicating typical development. Thus, FA appears to have had a toxic effect on the growth and development of *Drosophila*, and DHA was able to reverse this effect.

### 3.2 *Drosophila* mean body weight

**Figure 5:**
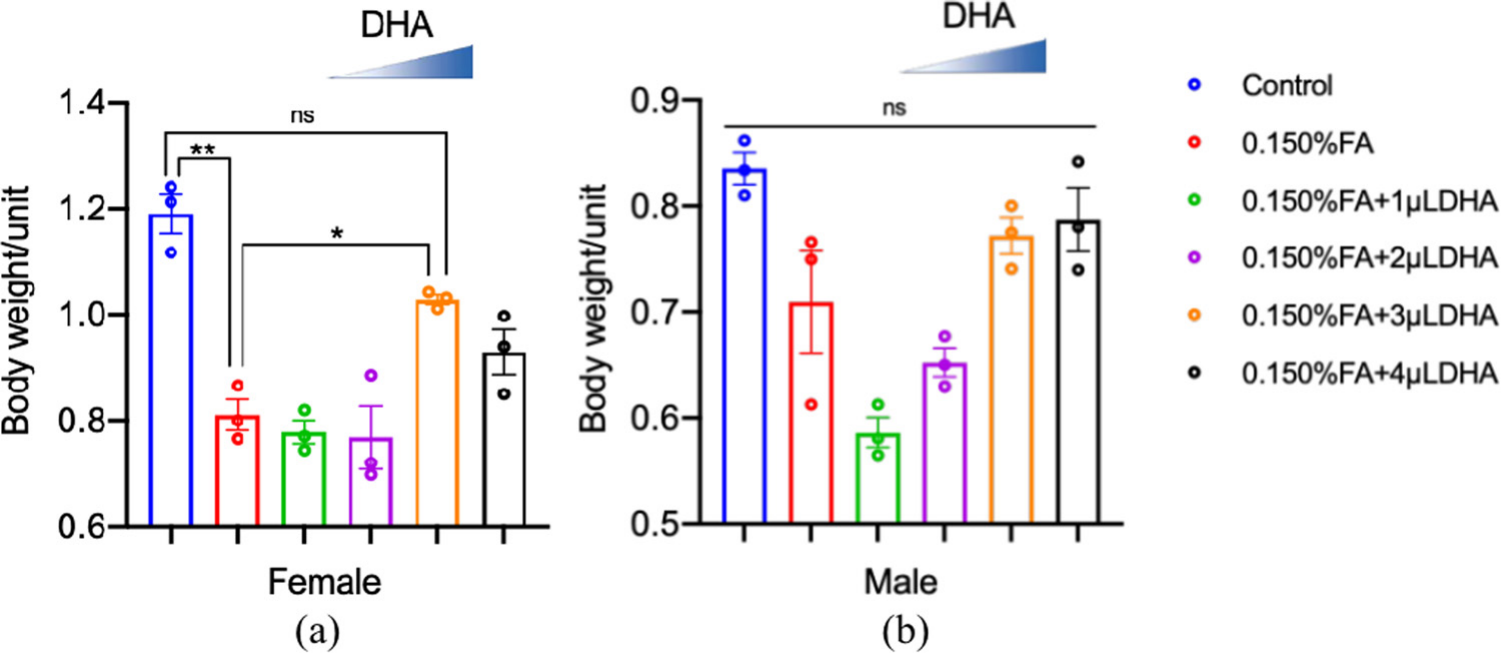
DHA reversed the FA-induced reduction of the female’s average weight. (a) The average weight of female was calculated from at least three independent experiments. (b) The average weight of male was calculated from at least three independent experiments. ns: no statistical significance, *P < 0.05, **P < 0.01, *t*-test, one-way ANOVA, Graphpad Prism 9.0.0.

We collected and weighed 20 3-day-old female and male adult flies in the F1 generation of each group. We then calculated the average weight of each female and male fly in each group (in mg). These results are presented in Figure 5. We found no significant differences in body weight among males. In females, the flies in the 0.150% FA group exhibited a significantly lower mean body weight compared with those in the control group (*p* < 0.01). Further, the fly body weight in the 0.150% FA + 3 µL DHA groups was increased compared with that in the 0.150% FA group (*P* < 0.05).

### 3.3 Lifespan

We assessed the lifespan of healthy adult *Drosophila* maintained in FA/DHA-added medium (for contrast with the FA and DHA-treated groups).

**Figure 6:**
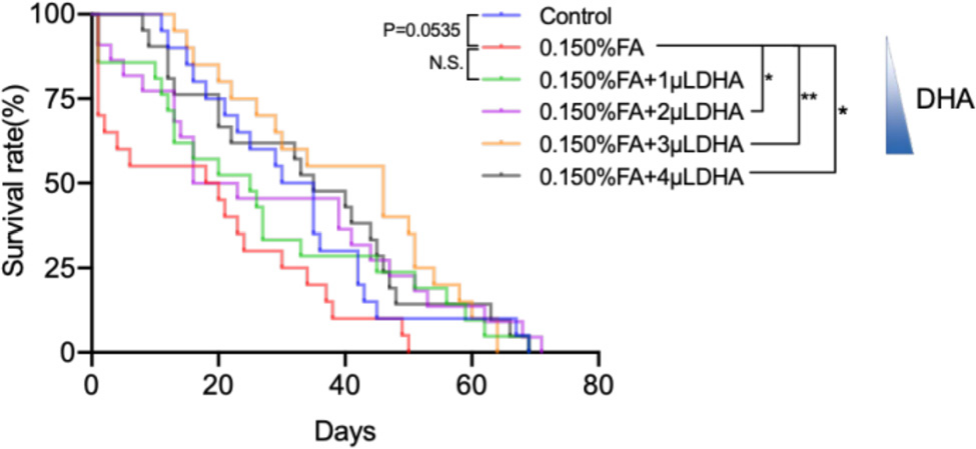
DHA can extend the lifespan of FA-induced *Drosophila*. The survival curve of the DHA gradient treatment of FA-induced *Drosophila* was measured from at least three independent experiments (males, *n* = 20). ns: no statistical significance; *P < 0.05, **P < 0.01, Log-rank test, Graphpad Prism 9.0.0.

To examine the life span of adult *Drosophila*, we calculated the time that had elapsed from the birth to the point at which all of the *Drosophila* in each group had died, as well as the number of flies that died per day. Until all flies died, the entire observation lasted for 69 days. The result is shown in Figure 6. The results showed that the 0.150% FA group’s lifespan was shorter than the control group (*P* = 0.0535). Compared with the *Drosophila* treated with 0.150% FA, DHA can extend the lifespan of 0.150% FA-induced *Drosophila*, such that the lifespan of the *Drosophila* in the 0.150% FA + 2 µL DHA, 0.150% FA + 3 µL DHA, 0.150% FA + 4 µL DHA group was significantly increased (*P* < 0.05).

### 3.4 Climbing assay

**Figure 7:**
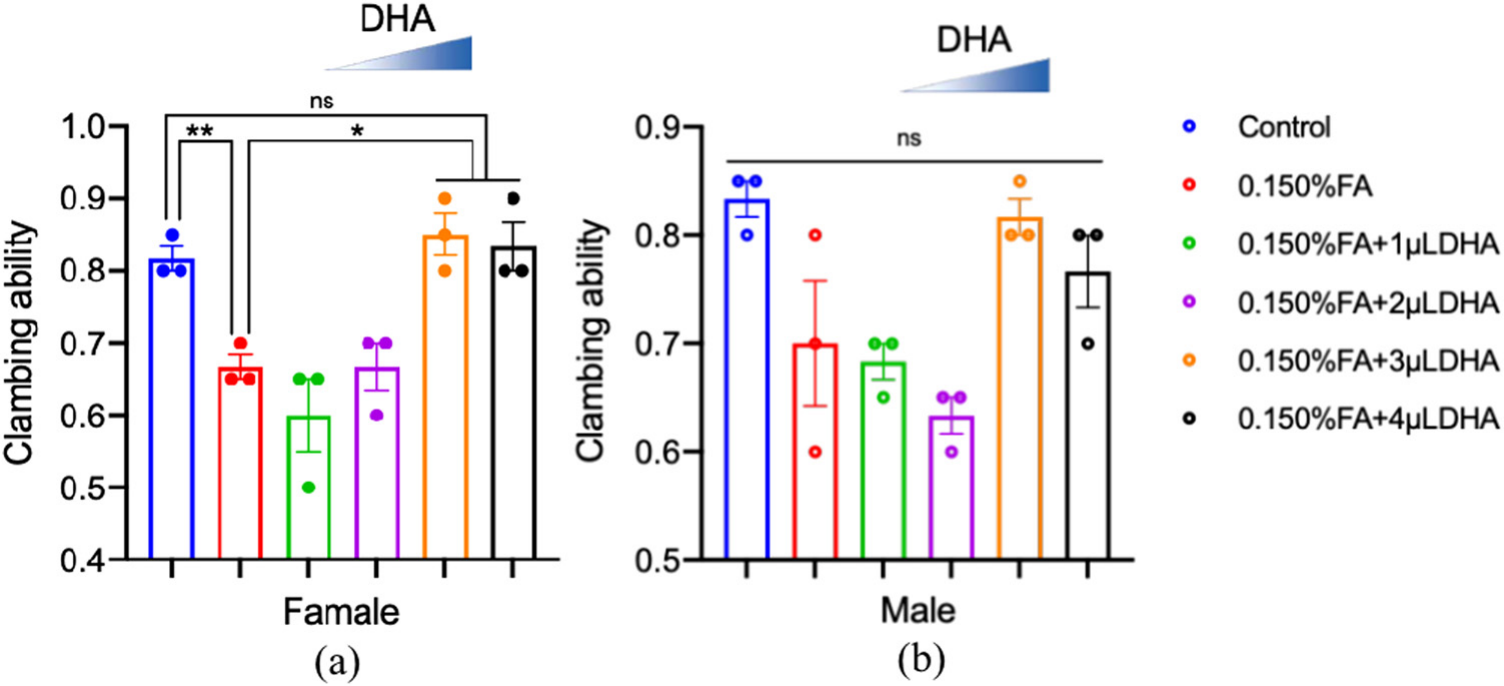
The impaired climbing ability induced by FA was rescued by DHA. (a) The climbing assay of female was carried out at 5 after eclosion with the indicated groups (n = 20); (b) The climbing assay of male was carried out at 5 after eclosion with the indicated groups (n = 20) ns: no statistical significance, *P < 0.05, **P < 0.01, *t*-test, one-way ANOVA, Graphpad Prism 9.0.0.

The eclosive female and male flies were cultured continuously for 6 days to determine their climbing abilities. To determine the effects of 0.150% FA or 0.150% FA + DHA at different concentrations on the locomotor ability of *Drosophila*, the results are shown in Figure 7.

For female *Drosophila*, the climbing ability of female flies in the 0.150% FA group was significantly lower than that in the control group (*P* < 0.01). Compared with the 0.150% FA group, when 0.150% FA was added with different concentrations of DHA, the climbing abilities of the female flies in the 0.150% FA + 3 µL DHA group and 0.150% FA + 4 µL DHA groups were significantly increased (*P* < 0.05). For the male *Drosophila*, compared with the control group, the climbing ability of the 0.150% FA group was slightly reduced, and DHA can slightly increase it.

### 3.5 Learning and memory

**Figure 8:**
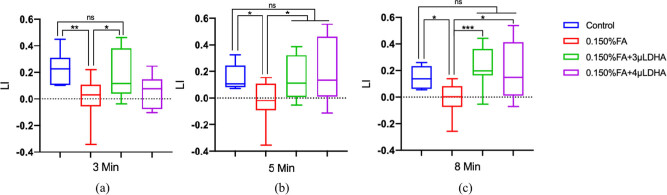
DHA protected against the toxicity of FA-induced learning and memory in 3rd larvae. LI was measured after training of 3(a), 5(b), and 8 min(c), respectively; each group repeated at least 5 times independently. ns: no statistical significance, *P < 0.05, **P < 0.01, ***P < 0.001, Mann–Whitney *U* test, Graphpad Prism 9.0.0.

To assess the effects of FA or FA with different concentrations of DHA on the *Drosophila* nervous system, “Pavlov’s Theory” holds that the establishment of classical conditioning requires the establishment of a causal association between conditioned and nonconditioned stimuli. We obtained a LI for each group of larvae and used LI to quantify the learning and memory ability of *Drosophila* larvae. The results are shown in Figure 8.

The results indicate that 0.150% FA reduced the learning and memory abilities of the larvae in 3 min, 5 min, and 8 min. The learning and memory abilities of the 0.150% FA + 3 µL DHA group and 0.150% FA + 4 µL DHA group are all significantly improved compared with the 0.150% FA group, except the 0.150% FA + 4 µL DHA group in 3 min.

### 3.6 Quantification of BDNF

**Figure 9:**
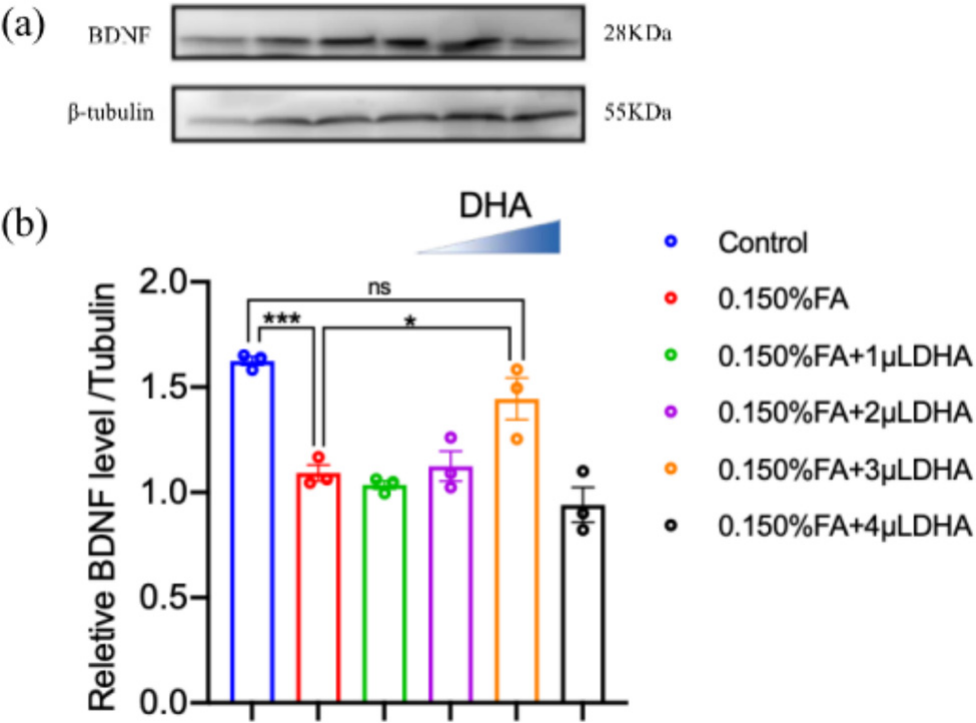
The expression level of BDNF in *Drosophila* brain in indicated groups. (a) Representative images of BDNF and β-tubulin quantification. (b) The statistics of the BDNF protein level. *P < 0.05, ***P < 0.001, one-way ANOVA, Graphpad Prism 9.0.0.

We assessed the protein quantification of BDNF in each group by western blot. The western blot data for BDNF and tubulin in the brains of the *Drosophila* from each group are shown in Figure 9(a). Image J shows the protein quantification level. The quantification level of BDNF protein in *Drosophila* brain tissue of each group was compared. The results of the analysis are shown in Figure 9(b).

The results indicate that the expression level of BDNF in the 0.150% FA group was significantly lower than that in the control group (*P* < 0.001), while that in the 0.150% FA + 3 µL DHA group was significantly higher than that in the control group (*P* < 0.01).

## 4 Discussion

### 4.1 Toxic effect of FA on *Drosophila* melanogaster

There have been many reports on FA-induced toxicity in animals. For instance, FA exposure was found to have toxic effects on the respiratory tract [36], central nervous system [37], skin [38], and eyes as well as carcinogenicity [39]. FA has also been associated with adverse reproductive and mutagenic effects [40]. In addition, exposure to gaseous FA could impair spatial learning and memory in mice, which could induce cognitive deficits [41]. In our *Drosophila* model, the results suggest that FA exposure can negatively impact on growth and development, reproductive ability, survival time, motor ability, and learning and memory function, which are consistent with the evidence in other animal models [42]. Although the detailed mechanism(s) related to FA-induced toxicity at the molecular level is unknown, it seems that the chromosome and DNA may have been damaged; consequently, the enzymes, proteins, and hormones have been altered, and thus, the reproductive, locomotive, and brain organs of FA-induced *Drosophila* have been dysfunctional. Hence, focuson the FA impact on DNA replication, transcription or protein translation is paid much attention.

### 4.2 Protective effect of DHA

DHA is a fatty acid with 22 carbon atoms and 6 double bonds. It plays an important role in the development of the nervous system [43]. For example, DHA is essential for the growth and functional development of the infant's brain and is also required for the maintenance of normal brain function in adults [44,45]. Even though there are many benefits of DHA on the nervous system, whether it might counteract the toxic effects of FA on various bodily systems is still unclear. In this experiment, we observed a particularly strong rescue of DHA on growth and development abnormality, reproductive disability, locomotive decreased, and learning and memory function altered based on FA-treated *Drosophila*. In terms of several outcome indicators such as body weight, climbing abilities, learning and memory, and the expression level of BDNF in the *Drosophila* brain, there was no significant difference between 0.150% FA + 3 µL DHA and the control group, which indicated that 3µL DHA had the best protection effect on *Drosophila*, and suggested that this concentration might be the optimal concentration for protecting *Drosophila* from the toxic effect of FA.

Given that BDNF is a widely present neurotrophic factor in the central and peripheral nervous system, which plays an important role in supporting the survival of existing nerve cells, promoting the generation of new nerves and synapses, and LTP related to the core of learning and memory [31,46], thus BDNF is regarded as a key factor for cognition and memory function [47]. In our *Drosophila* model, the level of BDNF (Figure 9) was significantly decreased in the 0.150% FA group when compared with the control group, which suggested the brain function has been altered. Surprisingly, when *Drosophila* ingested appropriate DHA, the BDNF level was significantly increased, which means that it could promote the expression of BDNF in the brain’s neurons, including mushroom neurons, which have a similar function with hippocampal neurons. Indeed, only using BDNF level as a marker for evaluating the function of the brain is not very convincing, and more studies are needed to confirm the neurotoxic effect of FA in the level of cell integrity and system functionality and the protective effect of DHA through investigation of factors involved with nerve cells growth and apoptosis and neural circuit.

In addition to BDNF, in future experiments, we will also envisage other indexes for evaluation: changes in nerve growth factor, insulin-like growth factor 2 (IGF-2), and vascular endothelial growth factor associated with neurologic development can be cited as evidence. LTP is considered to be the basic cellular mechanism of learning and memory [48]. Therefore, the activity and expression levels of neurotransmitters such as glutamic acid and gamma aminobutyric acid (GABA) involved in LTP formation as well as *N*-methyl-d-aspartate (NMDA) receptors, GABA receptors, and α-amino-3-hydroxy-5-methyl-4-isoxazolepropionic acid (AMPA) receptors can be detected. The mushroom body of *Drosophila* is the main place where learning and memory occur. The nerve damage caused by FA and the protection provided by DHA may be accompanied by the reduction or generation of axons in the mushroom body. Therefore, a single axon in the mushroom body can be detected by green fluorescent protein to reflect whether DHA improves neuronal synaptic formation in the mushroom body [49]. We can also observe the changes of factors involved in FA injury to detect the protect function of DHA. Studies have shown that FA can interfere with the NO/cGMP signaling pathway and then affect the concentration of cAMP, cGMP, and NO as well as the activity of NOS in the cerebral cortex, hippocampus, and brain stem of animals by causing oxidative stress damage in the brain [41]. In addition, FA can also interfere with the production of endogenous H2S in the hippocampus, leading to oxidative stress-mediated neuronal damage and eventually impairing learning and memory function [50]. Therefore, the concentrations of ROS, NO, cAMP, cGMP, NOS, and endogenous H2S in the brain tissue of *Drosophila* can be detected to confirm whether DHA directly participated in the injury pathway of FA to antagonize its neurotoxicity.

Altogether, these data indicate that DHA protects against the FA-induced toxicity of the reproductive, locomotive, and nervous systems.

In summary, our data provide evidence that DHA can protect against the toxicity induced by FA in the *Drosophila* model. Moreover, DHA shows neuroprotection against FA-caused disability in the learning and memory of larvae. In particular, much effort should focus on whether DHA counteracts the effects of FA-induced mice or rat animals, even monkeys.
